# A Clinical Decision Support System for the Prediction of Quality of Life in ALS

**DOI:** 10.3390/jpm12030435

**Published:** 2022-03-10

**Authors:** Anna Markella Antoniadi, Miriam Galvin, Mark Heverin, Lan Wei, Orla Hardiman, Catherine Mooney

**Affiliations:** 1 UCD School of Computer Science, University College Dublin, Dublin 4, Ireland; anna.antoniadi@ucd.ie (A.M.A.); lan.wei@ucdconnect.ie (L.W.); 2 FutureNeuro SFI Research Centre, RCSI University of Medicine and Health Sciences, Dublin 2, Ireland; hardimao@tcd.ie; 3 Academic Unit of Neurology, Trinity Biomedical Sciences Institute, Trinity College Dublin, Dublin 2, Ireland; galvinmi@tcd.ie (M.G.); mark.heverin@tcd.ie (M.H.); 4 Department of Neurology, National Neuroscience Centre, Beaumont Hospital, Dublin 9, Ireland

**Keywords:** artificial intelligence, Explainable AI, XAI, clinical decision support systems, CDSS, machine learning, explainability, ALS: Amyotrophic Lateral Sclerosis, MND, quality of life

## Abstract

Amyotrophic Lateral Sclerosis (ALS), also known as Motor Neuron Disease (MND), is a rare and fatal neurodegenerative disease. As ALS is currently incurable, the aim of the treatment is mainly to alleviate symptoms and improve quality of life (QoL). We designed a prototype Clinical Decision Support System (CDSS) to alert clinicians when a person with ALS is experiencing low QoL in order to inform and personalise the support they receive. Explainability is important for the success of a CDSS and its acceptance by healthcare professionals. The aim of this work isto announce our prototype (C-ALS), supported by a first short evaluation of its explainability. Given the lack of similar studies and systems, this work is a valid proof-of-concept that will lead to future work. We developed a CDSS that was evaluated by members of the team of healthcare professionals that provide care to people with ALS in the ALS/MND Multidisciplinary Clinic in Dublin, Ireland. We conducted a user study where participants were asked to review the CDSS and complete a short survey with a focus on explainability. Healthcare professionals demonstrated some uncertainty in understanding the system’s output. Based on their feedback, we altered the explanation provided in the updated version of our CDSS. C-ALS provides local explanations of its predictions in a *post-hoc* manner, using SHAP (SHapley Additive exPlanations). The CDSS predicts the risk of low QoL in the form of a probability, a bar plot shows the feature importance for the specific prediction, along with some verbal guidelines on how to interpret the results. Additionally, we provide the option of a global explanation of the system’s function in the form of a bar plot showing the average importance of each feature. C-ALS is available online for academic use.

## 1. Introduction

“Computer programs to assist with medical decision making have long been anticipated by physicians with both curiosity and concern”, stated an article from 1987, which described the state of the art in computer-based Clinical Decision Support Systems (CDSS) [1]. The authors argued that there was slow progress in the field, although it began almost 30 years prior [1]. Despite the fact that Artificial Intelligence (AI), and, more specifically, Machine Learning (ML) are currently offering increasingly potential for battling challenges in the field of medicine with their incorporation in CDSS, they are still surrounded by mistrust and scepticism.

ML has been used to develop CDSS that can process large amounts of existing information in order to assist and enhance clinical decision-making and, given the rapid advancements in ML techniques and hardware, as well as the availability of large datasets, they are considered by many as a promising asset to the clinical workflow [2,3]. CDSS have been reported to improve clinical practice by influencing patient safety, reducing the costs of healthcare [4], improving prescription practices, delivery of preventive and optimal treatment, and adherence to recommended standards and guidelines of care [5,6].

Low resource settings can benefit from CDSS not only because of the reduced costs but also due to the lack of experts and equipment in these settings. Generally, if designed appropriately, these tools could diminish the cognitive burden for clinicians, and save healthcare provider time by a significant amount (estimated around 65% [7]). Since a system that is 100% accurate does not exist, clinicians remain the main decision makers; ML-based systems have been called “*a second set of eyes to inspect a clinical case*” [8]. Thus, clinicians and CDSS can work in tandem towards improved healthcare outcomes for patients and their families.

Scepticism around CDSS usage arises mainly from concerns on safety, fairness, and usability [3,9,10,11,12]. This can be expected in an area, such as medicine and healthcare, where the risk for the individual that is affected by the outputs of the ML model is high. Explainability of the ML algorithms has been proposed as one of the solutions to this problem and has been in the spotlight of literature in recent years, with different techniques to explain the algorithm functionalities and predictions being developed under the umbrella of Explainable AI—or XAI [9].

An explainable system allows developers and clinical experts to validate the model [11] at all stages of development and testing and to identify potential biases. Additionally, the explainability of ML-based CDSS could enhance the clinicians’ trust towards the systems [13,14], while high accuracy alone is not sufficient [15], especially as it is often achieved by a model that is complex and opaque. For this reason, deep learning and ensemble techniques were deemed inappropriate for clinical decision support [16], as they were considered “black-box”. Clinicians may also need to explain the CDSS’s decision to patients and colleagues, and information on how it was produced should be available [15]. Finally, through sufficient explanations, under-reliance and over-reliance on the system could be averted if clinicians have an understanding of the system’s operation [11,17].

The Food and Drug Administration (FDA) moved towards establishing standards as a result of the growing interest in CDSS, and in January 2021 issued the “Artificial Intelligence/Machine Learning (AI/ML)-Based Software as a Medical Device (SaMD) Action Plan” (https://www.fda.gov/medical-devices/software-medical-device-samd/artificial-intelligence-and-machine-learning-software-medical-device, accessed on 23 November 2021). Moreover, in July 2021, the World Health Organisation (WHO) released AI Guidelines for Health [18], which offer six primary principles for the use of AI, including to protect autonomy and to ensure transparency, explainability, and intelligibility.

Autonomy indicates that, in medicine, decision-making should be conducted by humans rather than machines, while transparency, explainability, and intelligibility refers to improving transparency of the technology between developers and regulators as well as to medical professionals and patients affected by it. This reinforces the General Data Protection Regulation (GDPR) [19] interpretation that explainability is necessary and the data subjects that are impacted by a decision, should be able to be informed about the logic and significance of the automated decision via a CDSS. According to the GDPR [19], data subjects have “*the right not to be subject solely on automated processing*” (Article 22, Recital 71).

Attention should be paid to the fact that the right to obtain human intervention and not be subject solely to automated processing should not be interpreted in a way that trivialises the value of AI-based suggestions. We have seen that, in many cases, AI models, due to the technological advances and the volume and variety of data they can process, may perform similarly or even outperform medical experts [20,21,22,23].

As expressed by Lee [24]: “*The bottom line is that both AI and humans can make unique contributions to patient outcome prediction, and they should help each other to maximize predictive performance*”. To allow for this combined decision, XAI techniques can provide meaningful information on the logic behind the decision provided.

How meaningful the information provided is, may depend on the cognitive skills of the audience or the ML experience of clinicians. Although there is consensus among clinicians on the importance of explainability [15], different clinicians or different outcomes may require different explanations. Tonekaboni et al. [15] conducted interviews with clinicians to understand the aspects of explainability that can improve their trust in these systems.

In summary, the interviewed clinicians considered explainability as a means of justifying their clinical decision-making (e.g., to patients and colleagues) in the context of a model’s decision. The presentation of the model’s prediction should be designed so that they can understand and rationalise the predictions, and the CDSS should provide a level of transparency that allows users to validate model outputs with domain knowledge, as well as actionable steps they can take. They also considered that, although accuracy is important, the system needs to provide information in cases where the model may fall short, some expressing that lower accuracy could be acceptable provided that there is explanation around it.

Finally, the real-world application and success of the system was deemed crucial [15]. A systematic literature review on XAI-enabled and ML-based CDSS found that there was a small number of systems that focused on explainability as a key feature and that there was a limited number of user studies to evaluate this aspect of systems [9].

In this work, we present a prototype CDSS, called CDSS for ALS (C-ALS) for the prediction of quality of life (QoL) of people with Amyotrophic Lateral Sclerosis (ALS) or Motor Neuron Disease (MND). We found only one other CDSS to evaluate QoL in the literature, which was developed for head and oncology patients [25]. That system requires patients to answer questionnaires to calculate their QoL; however, we aimed for a system that will not require the input of any information directly from the patient. ALS is a rare neurodegenerative disease, characterised by the progressive death of upper and lower motor neurons, causing muscle atrophy and paralysis.

Due to the fact that ALS is currently incurable, treatment is mostly palliative and aims to alleviate symptoms and improve QoL [26,27]. QoL is regarded as a basic goal of health and social care. It is determined by health-related and non-health related factors [28]. The QoL data in this study were collected by the standardised measure McGill QoL questionnaire [29].

C-ALS was created with a view to helping healthcare practitioners identify people with ALS (PALS) at risk of low QoL sooner and to facilitate the discussion around QoL and potential supports with them and with colleagues [30,31,32]. Our system was designed so that it can retrieve information from connected electronic sources (e.g., the National ALS Registry) and can also take input from the clinician at the point of care. The risk of low QoL is displayed in the form of a probability followed by an explanation. The first version of our system’s explanation was evaluated by healthcare professionals involved in the treatment of ALS in the National ALS/MND multidisciplinary clinic (MDC) at Beaumont Hospital, Dublin, Ireland. This short study was followed by an updated version of our CDSS.

Personalized medicine is about optimizing treatment according to unique patient characteristics as opposed to providing the same treatment to people with the same condition, in this case, ALS. Personalized medicine may be based on a patient’s genomics, genetics, medical status, personal circumstances, and care preferences and holds great potential in healthcare over “one-size-fits-all” approaches [33]. Although it has mostly been applied to therapeutic or preventive interventions, it generally aims to restore patient health with maximized QoL [34].

The proposed CDSS aims to inform QoL-targeted decisions to personalize the support provided to PALS or their caregivers, including but not limited to therapeutic interventions. The decision makers of these interventions are the members of the multidisciplinary caring team, and thus, to achieve this goal, the CDSS needs to be explainable and informative to them.

## 2. Materials and Methods

### 2.1. C-ALS Model

The model that was used for the development of C-ALS is described in [30]. The features used in the analysis contained patient and primary caregiver demographic information, use of health services, financial support, social status, aids and appliances received, expenses for treatment, clinical attributes of the patient, and caregiving duties of the caregiver. In order to select a subset of the most relevant features, we applied an ensemble of six different feature selection methods for the elimination of biases in the process [32].

This step was conducted in R (http://www.r-project.org/, accessed on 4 September 2020) using the “EFS” (Ensemble Feature Selection) package [35]. From this methodology, we identified the 50 most important features for the prediction of QoL, and then we worked on developing models consisting of smaller subsets of features. The “best” model in terms of accuracy was one that used three of these features. It was developed using the Extreme Gradient Boosting algorithm (XGBoost) [36], a popular implementation of the gradient tree boosting method [37].

**Figure 1:**
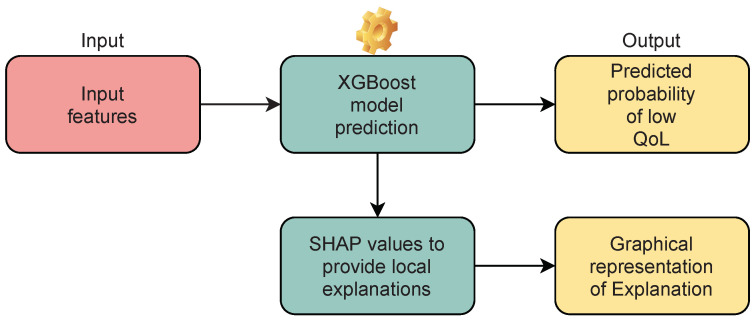
Architecture of C-ALS (input, process, and output). The input feature space consists of the three features that were identified to be predictive of QoL (the patient’s age at disease onset, the primary caregiver’s employment status before the onset of their caregiving duties, and the patient’s ALSFRS-R score for orthopnoea). The three features are used by the XGBoost model to predict the outcome in the form of a probability, while SHAP is used to provide local explanations for the specific prediction in the form of a graphical representation.

The output is explained with the help of a *post-hoc* XAI technique, namely SHAP (SHapley Additive exPlanations) [38]. SHAP values are a unified method of other XAI methods to measure feature importance after the model is trained and can provide both local explanations for a specific prediction as well as global explanations to give an overview of the model’s logic. Although XGBoost can plot the global feature importance, we think that local explanations on the specific patient are the most informative for personalised decisions; therefore, SHAP assisted in this task. The architecture of the CDSS is presented in Figure 1, while more detailed information can be found in [30,31].

We selected a model with three features that were identified as predictive of patient QoL in our previous work [30,31]. Two of those predictors need to be collected only once, while the third is routinely collected at the patients’ visit to the clinic. The first two features are the patient’s age at disease onset, and the primary caregiver’s employment status before the onset of their caregiving duties, while the latter is the patient’s ALSFRS-R (ALS Functional Rating Scale-Revised) score for orthopnoea.

The possible values for the ALSFRS-R score range between (0) and (4), where (4) corresponds to no breathing problems when lying flat; (3) some difficulty sleeping at night due to shortness of breath, but does not routinely use more than two pillows; (2) needs extra pillows in order to sleep (more than two); (1) can only sleep sitting up; and (0) unable to sleep.

The caregiver’s employment status is a categorical variable where: (1) represents working for payment or profit; (2) looking for first regular job; (3) unemployed; (4) student/pupil; (5) looking after home/family; (6) retired from employment; (7) unable to work due to permanent sickness or disability; and (8) other. The outcome is a probability (a value between 0 and 1); the closer this value is to 0, the lower the risk of poor QoL, while the closer it is to 1, the higher the risk.

### 2.2. Implementation of C-ALS Prototype

**Figure 2:**
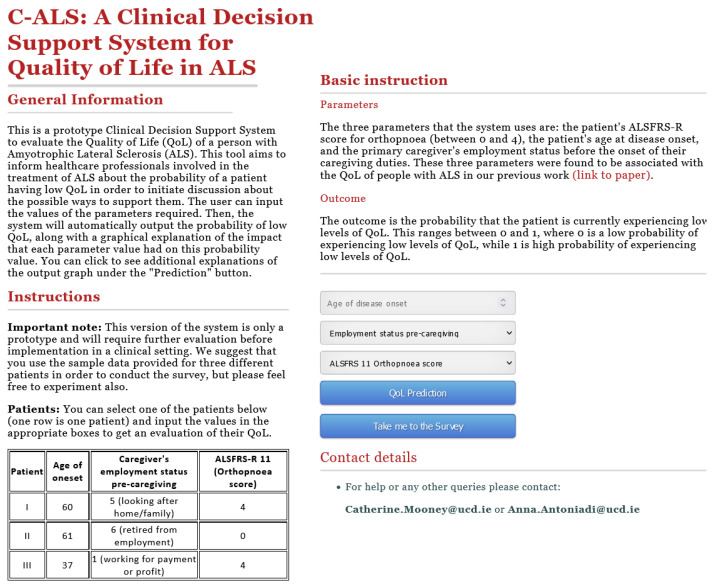
Screenshot of the first page of C-ALS. The first page describes the CDSS and three exemplar patients and allows the user to input the feature values to obtain a prediction. The prediction along with explanations opens in a new window.

Based on the previously described model, we developed a CDSS interface. A screenshot of the first page of the C-ALS interface is shown in Figure 2; however, for better readability or to interact with the system, we advise that you visit http://lisda.ucd.ie/C-ALS, accessed on 7 February 2022. On the first page, we provide some information on the system: the aim, input, and output, along with some exemplar patients that we suggest the users to experiment with. On the bottom right of that page, the user can input the values and click to make a QoL prediction.

The users can fill in the values of the three parameters: the patient’s ALSFRS-R score for orthopnoea (between 0 and 4), the patient’s age at disease onset, and the primary caregiver’s employment status before the onset of their caregiving duties; these are described in the top right of the page, along with a reference to our related work [30]. The output is also explained on this page, prior to the QoL prediction. We clarify that it will be presented as a probability that the patient is currently experiencing low levels of QoL: (0) is a low probability of experiencing low QoL, while (1) is a high probability of experiencing low QoL. The user can then insert the parameter values in the boxes below; the employment status of the caregiver and the ALSFRS-R score for orthopnoea can be selected from a drop-down menu.

#### 2.2.1. First Version of C-ALS and User Evaluation

**Figure 3:**
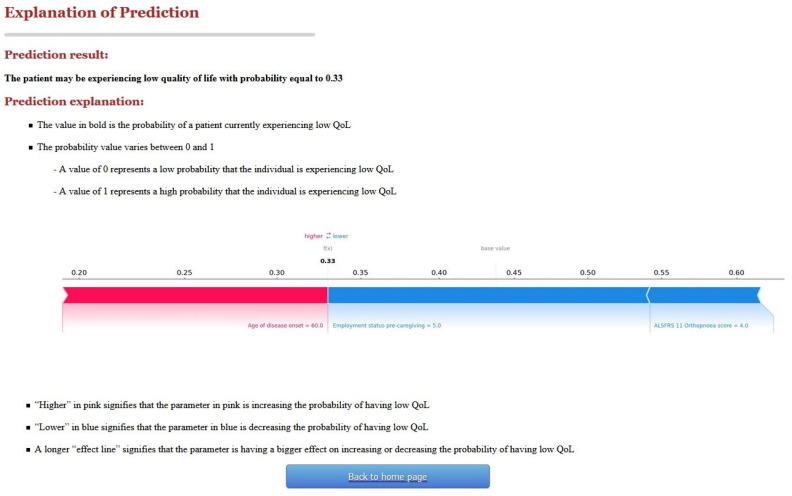
Screenshot of a prediction explained by version 1 of C-ALS.

Figure 3 shows the prediction results and the explanation of that prediction. We chose one of the exemplar patients as an example: the patient’s age of disease onset is 60 years old, the caregiver’s employment status pre-caregiving is 5 (looking after home/family), and the ALSFRS-R score for orthopnoea is 4. The prediction result shows that the patient may be experiencing low QoL with probability equal to 0.33.

The probability value in this version of the CDSS is followed by a local explanation that is based on SHAP values, visualised with an additive force layout (using a forceplot from the SHAP library in Python (https://www.python.org/, accessed on 21 August 2020). The system shows the features that affected the prediction along with the value that each feature had and its effect on the output.

It also provides a general interpretation of the graph as follows: “Higher” in pink signifies that the parameter in pink is increasing the probability of having low QoL; “Lower” in blue signifies that the parameter in blue is decreasing the probability of having low QoL; A longer “effect line” signifies that the parameter is having a greater effect on increasing or decreasing the probability of having low QoL. In this particular example, the user could infer that the aspect that mostly affected this outcome was the caregiver’s employment. While this patient’s age at disease onset was related to low QoL, the caregiver’s employment status along with the patient’s orthopnoea score had a greater effect on eventually decreasing the probability of this patient experiencing low QoL.

#### 2.2.2. User Evaluation

The first version of C-ALS was presented to healthcare professionals along with a short survey on its explainability. Ethical approval for this work was granted by the University College Dublin Human Research Ethics Committee (LS-E-21-132-Mooney). Participants were contacted via email and provided informed consent before participating in the survey, while their data were collected in an anonymous manner. A total of 28 healthcare professionals were invited to take part in the survey. These healthcare professionals constitute the entire multidisciplinary clinical team that make up the National ALS/MND multidisciplinary clinic (MDC) in Beaumont Hospital, Dublin, Ireland, and they were asked to participate in the study in June 2021. This clinic sees approximately 80% of all incident cases of ALS in the Republic of Ireland on at least one occasion and a large subset of these cases on a recurrent basis every 6–8 weeks.

Eight out of 28 healthcare professionals responded to the survey, a 29% participation rate. This participation rate is close to the one observed by Koning et al. [39] in their study on Neurosurgery Survey Response Rates to evaluate survey fatigue during the COVID-19 pandemic. The authors found that most studies were also multiple choice and disseminated primarily by email, and there was a decrease in response rates (34.5 vs. 51.0%, *p* < 0.001) compared to pre-COVID-19 surveys.

An electronic questionnaire was sent to participants, and it consisted of 12 multiple choice or Likert scale questions, where the users were asked their views on explainability. In the 5-point Likert scale, 1 was “strongly disagree” and 5 was “strongly agree”. There was also an open-ended question for suggestions on the explainability, additional features for the CDSS or other.The survey questions were based on the study by Tonekaboni et al. [15] that was described in the Introduction Section. The survey results are presented by raw counts and percentages, while the answers to the open-ended questions are conveyed as expressed by the respondents.

## 3. Results

### 3.1. User Evaluation

We first started with some preliminary questions and found that none of the respondents had used a CDSS before. If given the option, all users would rather have a prediction along with explanations over an outcome without explanations. Almost all respondents (seven out of eight, or 87.5%) said they would not use a CDSS that did not provide any explanations, while one person said they might use it if they knew that the system was correct 80% of the time. Table 1 shows the questions and possible answers containing the raw number and percentage of respondents that gave a particular score to each of the Likert questions. Three participants responded to the open-ended question on additional ways to improve the system’s explainability or other suggestions:

1. Respondent 1: “Maybe another sentence or categorisation after the probability in bold? e.g., mild/moderate/high risk of low QoL? You could also add the three variables into a short narrative to explain their influence on the patient’s QoL, e.g., employment status pre caregiving had the greatest impact etc.”.
2. Respondent 2: “Employ clinicians to advise out terminology and output”.
3. Respondent 3: “The areas assessed seems very narrow”.

### 3.2. Second Version of C-ALS

**Figure 4:**
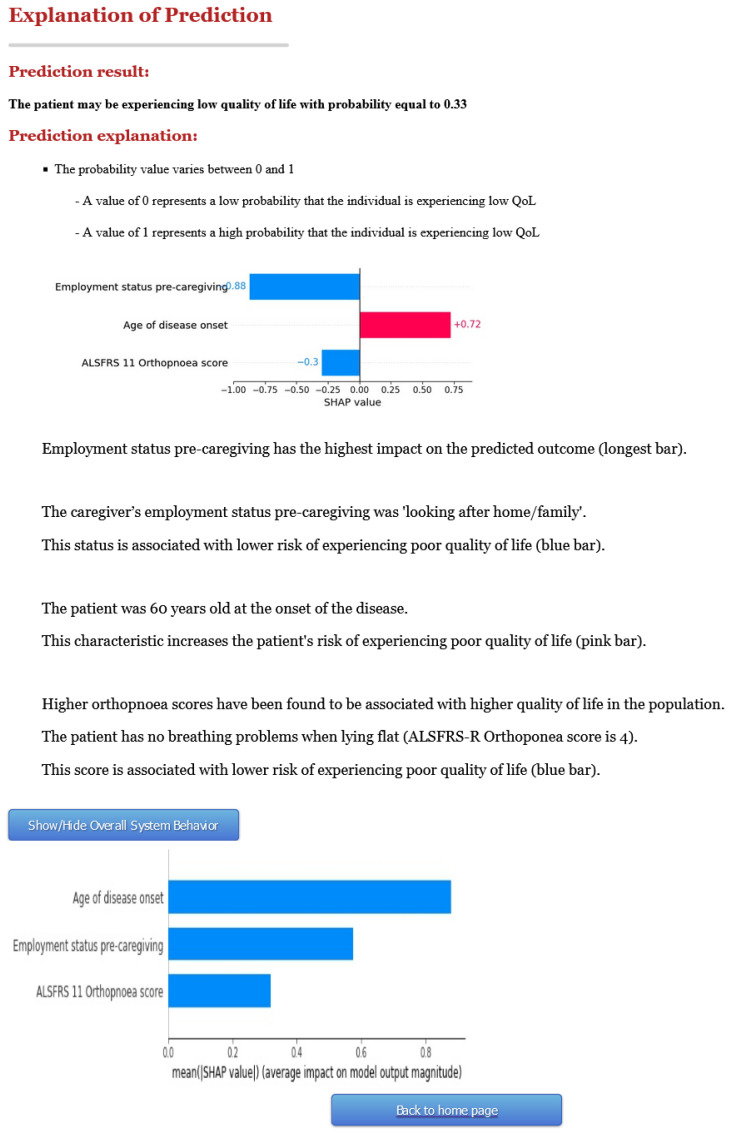
Screenshot of a prediction explained by version 2 of C-ALS.

Following the short user survey, we found that there was some uncertainty around the users’ understanding and justifying of the CDSS explanations, as well as the user’s trust of model predictions. Based on this feedback, as well as the suggestion of providing a narrative to explain the variables’ influence on the prediction, we updated C-ALS. A screenshot of the second version is presented in Figure 4. The first page remains the same for both versions, and thus Figure 4 displays only the explanation page, using the same exemplar patient as Figure 3.

First, we changed the graph of the local explanation from a forceplot to a bar plot. We verbally describe each of the features and its impact on the prediction, in context with the graph. We included some information about the overall impact that high orthopnoea scores have on the output, as well as the impact of a specific category of employment status (i.e., “working for payment or profit) when its value appears. We changed the phrasing of the explanations from “increasing/decreasing the probability” to “increasing the risk/ being associated with a lower risk of experiencing poor QoL”. Finally, we give the user the option (through a “Show/Hide Overall System Behavior” button) to view a bar plot of the global explanation of the model’s important features.

## 4. Discussion

According to studies by Tonekaboni et al. [15] and Mitchell et al. [40], the variables that derived the decision of the model constitute critical information that should be provided to clinicians. C-ALS, a prototype CDSS that aims to inform healthcare practitioners on the QoL of PALS, provides this information for each individual prediction in the form of local SHAP explanations. The prediction is presented in the form of a probability, and both prediction and visual representationare enhanced by verbal explanations.

We also included the option to view a global feature importance bar plot for comparison. C-ALS is currently available online (http://lisda.ucd.ie/C-ALS, accessed on 7 February 2022) and intended for academic use. When asked about the usefulness of a CDSS that evaluates QoL in ALS, healthcare professionals expressed a medium to high interest. This could depend on the role that each participant has in the care of PALS; however, we believe that the development of such a system could raise awareness regarding the importance of evaluating QoL in terminal conditions in general [41,42] and ALS in particular [43].

Although Tonekaboni et al. [15] found that presenting important features can draw the attention of clinicians to specific patient characteristics and allows them to determine how to proceed, in our study, responses were more neutral regarding actionable steps they could take. This may be expected considering the fact that the first version of our CDSS was moderately successful in explaining its outputs. This may have been resolved to some extent in the updated version, where the different graph and more descriptive verbal explanations could have led to higher scores in the questions relating to rationalising, understanding and explaining predictions.

Future work could also follow the suggestion made by some of the clinicians in the study by Tonekaboni et al. [15] of presenting similar samples to their patients to understand what actions were taken in those cases. The suggestions that followed in the open-ended question showed some scepticism regarding the features that the CDSS decisions are based on (Respondent 2) in terms of their number, while the clinical relevance of the features was not reported as an issue here. Validation on more healthcareprofessionals is necessary to confirm the accuracy of this model; we also created a five-feature model with similar accuracy; however, we selected the simpler model for this prototype and this user study [30,31]. A concern regarding the terminology and output was expressed (Respondent 3) that suggested closer collaboration with members of the clinical team on the use of appropriate terminology.

The final system needs to be designed in a way so that all users can understand and rationalise the output, as relevant for them. With MLand AI becoming increasingly promising in the field, the gap of trust and acceptance of CDSS could be narrowed if some computer-based training [12] or introductory ML knowledge is provided to healthcare professionals [44]. The suggestion for designing such a curriculum is to aim for ML literacy rather than proficiency and to make trainees comfortable with the concept and tools [44]. Additionally, the implementation of appropriate protocols for implementation and use may ease concerns related to the use of technology in healthcare [12].

As discussed in the introduction, there is a distinct lack of user studies for XAI-enabled AI-based CDSS. One study conducted on a CDSS for child health, evaluated by six clinicians, found that the CDSS information elements that were rated as the most important by clinicians were the following: (a) the information the system used to make this diagnosis; (b) the information that supports this diagnosis; (c) the information that contradicts this diagnosis; (d) other diagnoses that are conceivable based on the case information; (e) how certain the system is of this diagnosis; (f) the information that would increase the certainty of this diagnosis; and (g) the performance of the system for other, similar cases [45].

While our system provided information in agreement with (a) and (b), it lacked the remaining elements, which could be reason for the lack of some clinician engagement with our system. Six experienced otolaryngologists evaluated the CDSS presented in the work by Müller et al. [46] and all physicians emphasized the importance and clinical relevance of visual explanation and guidance in a CDSS, while valued structured, clear, and familiar presentation of all evidence items, which resembles their regular decision-making process.

The system by Müller et al. [46] was an interactive one that provided extensive information, which was displayed by the system, many times in the form of on-demand or hover-on evidence items.

The structure of this CDSS is also something to consider in the future while improving our own system’s visual and verbal explanations, and enriching C-ALS with more information that will not overwhelm the user. Finally, Lamy et al. [47] recruited 11 medical experts to evaluate their CDSS, who were enthusiastic regarding the interactive visual approach of the system. They particularly liked the qualitative approach that links system recommendations with patient characteristics in a manner close to their own logic, while their decision was guided by the case closest to the query.

In addition to explainability, a variety of recommendations for CDSS development and incorporation in clinical practice has been discussed in the literature. First, a systematic review of trials that aimed to capture features that have led to the success of CDSS reported that decision support had been computer-generated and was provided automatically and as part of the clinical workflow at the time and location of decision making [5].

The authors of this study also ran their own experiment and found improved effectiveness when clinicians had to present the reasons for non-compliance with the CDSS recommendations, when they received feedback based on their compliance, and when the results of the system were presented to both clinicians and patients [5].

Garg et al. [48] mentioned that the performance of practitioners was improved when they were prompted automatically by the CDSS rather than when they were required to activate the system, which is in agreement with the work of Kawamoto et al. [5]. Pathologists’ ability in error detection can be enhanced with the use of a CDSS that incorporates appropriate User Interface design choices to display them [8].

In agreement with Kawamoto et al. [5], Fiks [49] found that fitting CDSS into clinical workflows is an important factor, along with emphasis on simplicity and usability of the system. Clinicians also expressed a preference of systems that use existing information (e.g., from Electronic Health Records) without requiring the entry of additional information [49]. Generally, we can see that input from practising clinicians is crucial for the development and integration of a CDSS into a clinical workflow as their needs need to be addressed [49,50]. Moreover, this can create a more receptive environment for the CDSS and reduce doubts about the quality of the system’s recommendations [49].

### Limitations and Future Work

C-ALS is a prototype system that requires further research to improve the interface and explanations for clinicians. Additionally, it is based on a ML model that was built using a small sample size (due to the rarity of the disease) on an Irish population. As a result, our intent is to evaluate the CDSS on both healthcare professionals and patients in the future and to collect data from an EU cohort to examine a potential ethnic/cultural difference in QoL prediction, similarly to the study by Du et al. [51]. This will allow us to expand the scope of our CDSS beyond the Irish population. Subsequently, we aim that our previous work in predicting the QoL [52] and burden [53] of caregivers of PALS will be incorporated in the CDSS to provide a more holistic evaluation and support of those affected by the condition.

We are also aware of the user study’s limitations due to the small number of participants and narrowed scope of questions partly to reduce the survey time and partly to ensure anonymity. Yet this study provided preliminary indications on some important points that allowed us to improve our system’s explainability. The COVID-19 pandemic has affected clinicians’ time and survey participation [39].

The time that clinicians have available is generally limited; even pre-COVID-19 where survey participation was higher, it was still only around 51%. This reflects the potential of a CDSS to ensure the evaluation of patients’ QoL as part of their in-clinic treatment without additional workload on clinicians or even patients. QoL is an aspect that is mostly assessed in clinical research rather than during treatment; therefore, this enhances its prospective benefit.

It is possible that some of the clinicians who decided not to take part in our study, as well as some of those who did, do not find a CDSS that assesses patient QoL useful. This is expected according to Liberati et al. [50] who studied doctors, nurses, and other actors that play an important role in shaping the structural and political underpinning of CDSS adoption, such as information technology (IT) staff and members of the hospital board of directors. There are clinicians that do not consider CDSS as useful working tools, and others who consider them a “potential hindrance to the exercise of clinicians’ judgment”, who usually lack familiarity with information technologies.

In our study, although we did not test familiarity with information technologies, none of the participants had prior CDSS experience. This, in addition to the fact that there was an apparent disconnection between computer scientists and healthcare professionals in our work, is the main drawback, and thus we believe that future developers of CDSS should first ensure that connection by involving potential users early in the study. For the future acceptance of CDSS, it has also been suggested that senior leaders should raise awareness of the functions of the CDSS and its transparent inter-disciplinary use, while adaptation to the needs of different users and workflows is necessary on behalf of the system itself [50].

## 5. Conclusions

Trust towards CDSS comes from both accuracy and explanations; although some clinicians might be willing to accept lack of explanations for high accuracy, or lack of accuracy that is followed by explanations, the general conclusion from this and other studies is that they are both crucial for clinician trust and acceptance, especially when clinicians have no prior experience with CDSS. SHAP values may be understood by clinicians but not in the necessary level or at least not without additional verbal explanations.

A first user survey already allowed us to improve C-ALS, while a closer collaboration with healthcare professionals and patients could lead to even better and more explainable outputs and a more usable CDSS. Healthcare professionals could also advise on actionable steps that can be taken based on the produced outcome to enhance the system’s usefulness and, hence, usability. C-ALS is intended for academic use; however, we hope for its future incorporation into clinical practice. This study is the beginning of a collaboration between computer scientists and clinicians. With clinicians becoming more familiar with CDSS, this is a promising start, although there is still progress to be made.

## Figures and Tables

**Table 1:** Overall CDSS evaluation. Responses to 5-point Likert questions. 1: strongly disagree, 5: strongly agree.

Question	1	2	3	4	5
Would you use a CDSS that may fall short in accuracy (i.e., sometimes make a wrong prediction) provided that an explanation is provided?	0 (0%)	2 (25%)	6 (75%)	0 (0%)	0 (0%)
Would you find a CDSS that assesses the QoL of a patient with ALS useful for your decision-making regarding the patient’s and caregiver’s support provision?	0 (0%)	1 (12.5%)	4 (50%)	2 (25%)	1 (12.5%)
Regarding our CDSS, would the provided output and explanation help you justify your clinical decision-making (e.g., to patients and colleagues)?	0 (0%)	1 (12.5%)	4 (50%)	3 (37.5%)	0 (0%)
Does the visual representation of the CDSS output help you understand the predictions?	0 (0%)	0 (0%)	3 (37.5%)	5 (62.5%)	0 (0%)
Does the visual representation of the CDSS output help you rationalise the predictions?	1 (12.5%)	0 (0%)	2 (25%)	5 (62.5%)	0 (0%)
Does the explanation provided add towards your trust of model predictions?	0 (0%)	0 (0%)	5 (62.5%)	3 (37.5%)	0 (0%)
Does the explanation provided help you decide on actionable steps you can undertake?	0 (0%)	1 (12.5%)	5 (62.5%)	2 (25%)	0 (0%)

## Data Availability

No additional data are available.

## Abbreviations

The following abbreviations are used in this manuscript:

AI	Artificial Intelligence
ML	Machine Learning
CDSS	Clinical Decision Support Systems
XAI	Explainable AI
ALS	Amyotrophic Lateral Sclerosis
QoL	Quality of Life
PALS	People with ALS
